# Non-coding RNA Activated by DNA Damage: Review of Its Roles in the Carcinogenesis

**DOI:** 10.3389/fcell.2021.714787

**Published:** 2021-08-13

**Authors:** Soudeh Ghafouri-Fard, Tahereh Azimi, Bashdar Mahmud Hussen, Atefe Abak, Mohammad Taheri, Nader Akbari Dilmaghani

**Affiliations:** ^1^Department of Medical Genetics, Shahid Beheshti University of Medical Sciences, Tehran, Iran; ^2^Men’s Health and Reproductive Health Research Center, Shahid Beheshti University of Medical Sciences, Tehran, Iran; ^3^Department of Pharmacognosy, College of Pharmacy, Hawler Medical University, Erbil, Iraq; ^4^Phytochemistry Research Center, Shahid Beheshti University of Medical Sciences, Tehran, Iran; ^5^Urology and Nephrology Research Center, Shahid Beheshti University of Medical Sciences, Tehran, Iran; ^6^Skull Base Research Center, Loghman Hakim Hospital, Shahid Beheshti University of Medical Sciences, Tehran, Iran

**Keywords:** NORAD, lncRNA, cancer, expression, carcinogenesis

## Abstract

Long intergenic non-coding RNA 00657 (LINC00657) or “non-coding RNA activated by DNA damage” (NORAD) is an extremely conserved and copious long non-coding RNA (lncRNA). This transcript has pivotal role in the preservation of genome integrity. Several researches have appraised the role of NORAD in the evolution of human cancers with most of them indicating an oncogenic role for this lncRNA. Several miRNAs such as miR-199a-3p, miR-608, miR−155−5p, miR-590-3p, miR-495-3p, miR-608, miR-202-5p, miR-125a-3p, miR-144-3p, miR−202−5p, and miR-30a-5p have been recognized as targets of NORAD in different cancer cell lines. In addition, NORAD has interactions with cancer-related pathways, particularly STAT, TGF-β, Akt/mTOR, and PI3K/AKT pathway. Over-expression of NORAD has been related with poor clinical outcome of patients with diverse types of neoplasms. Collectively, NORAD is a prospective marker and target for combating cancer.

## Introduction

Long intergenic non-coding RNA 00657 (LINC00657) or alternatively named as “non-coding RNA activated by DNA damage” (NORAD) is an extremely conserved and copious long non-coding RNA (lncRNA; Lee et al., 2016). This transcript has a crucial role in the conservation of genome stability since its inactivation results in striking aneuploidy in formerly karyotypically normal cells (Lee et al., 2016). This function of NORAD is exerted through sequestering Pumilio RNA Binding Family Members (Lee et al., 2016). In addition, NORAD has functional interactions with an element of DNA-damage system namely RNA Binding Motif Protein X-Linked (RBMX). NORAD regulates the capacity of RBMX to construct a ribonucleoprotein complex which encompasses a number of proteins such as topoisomerase I (Munschauer et al., 2018). Depletion of NORAD results in high rate of chromosome segregation impairments, abridged replication-fork speed and changed cell-cycle movement (Munschauer et al., 2018). Due to the critical role of NORAD in the maintenance of genome stability and cell cycle progression, it is not surprising that dysregulation of this lncRNA leads to cancer. Therefore, several studies have appraised the role of this NORAD in initiation or progression of diverse types of malignancies. In the current review, we describe the role of NORAD in the evolution of human cancers based on the conducted experiments in cell lines, animal models and human subjects.

## Cell Line Studies

Expression of NORAD has been down-regulated in endometrial cancer cells. Forced up-regulation of this lncRNA suppressed growth of endometrial cancer cells and enhanced their apoptosis. Such effects have been exerted through NORAD binding with the anti-apoptotic protein Far Upstream Element Binding Protein 1 (FUBP1). Interaction between NORAD and FUBP1 has been shown to decrease nuclear localization of this anti-apoptotic protein, releasing the pro-apoptotic gene promoters from FUBP1 occupation and enhancing apoptosis in these cells (Han et al., 2020). A single study in colorectal cancer cells showed down-regulation of NORAD. Forced over-expression of NORAD reduced cell viability and invasiveness of these cells while enhanced cell apoptosis. This lncRNA has increased expression of Calpain 7 (CAPN7) and suppressed activity of PI3K/AKT pathway (Lei et al., 2018). However, two other studies in colorectal cancer cells reported the role of NORAD in increasing cell viability, proliferation, migration and invasion while inhibiting apoptosis (Wang et al., 2018; Zhang et al., 2018). Other studies in diverse cancer cell lines also supported the oncogenic role of this lncRNA. For instance, in ovarian cancer cells, over-expression of NORAD has been correlated with down-regulation of miR-199a-3p. NORAD silencing could suppress proliferation, invasiveness, migratory potential, and epithelial-mesenchymal transition (EMT) of these cells. Functional studies confirmed the direct interplay between NORAD and miR-199a-3p (Xu C. et al., 2020). Besides, NORAD up-regulation has enhanced migration and invasion of hepatocellular carcinoma cells through sponging miR-202-5p, which acts as a tumor-suppressor miRNA through the TGF-β pathway (Yang et al., 2019a). The functional effect of NORAD in activation of TGF-β signaling has also verified in breast cancer cells (Zhou et al., 2019). In lung cancer cells, NORAD promotes EMT-like characteristics through activation of TGF-β signaling. In this type of cancer, importin β1 has been found to be a binding partner of NORAD. NORAD silencing has inhibited the physical interaction between importin β1 with Smad3 to some extent, thus blocking amassment of Smad complexes in the nucleus following induction with TGF−β. Therefore, NORAD facilitates the interaction between importin β1 and Smad3 to enhance nuclear amassment of Smad complexes following exposure to TGF-β (Kawasaki et al., 2018). Lentivirus-mediated silencing of NORAD in epithelial cancer cells has inhibited proliferation, reduced chemoresistance and attenuated cell cycle progression. These roles are exerted through acting as a molecular sponge for hsa-miR-155-5p (Tong et al., 2019). In cervical cancer cells, NORAD enhances expression of SIP1 to increase cell proliferation, invasiveness and EMT. These effects are due to sponging miR-590-3p (Huo et al., 2018). In neuroblastoma, in addition to enhancement of cell proliferation and invasion, NORAD increases doxorubicin resistance possibly through suppression of apoptosis and autophagy. These effects are exerted through miR-144-3p/HDAC8 axis (Wang et al., 2020). In osteosarcoma cell lines, NORAD regulates cancer cell features via acting as a molecular sponge for hsa-miR-199a-3p (Wang et al., 2019). Another study has shown that transcription of NORAD is suppressed by the YAP/TAZ-TEAD complex, a transducer of Hippo pathway. NuRD complex also facilitates transcriptional silencing of NORAD through this route. NORAD exerts effective suppressive impact on migration and invasion of neoplastic cell lines, and blockage of NORAD expression contributes in the pro-migratory and invasive impacts of the YAP pathway. Functionally, NORAD uses its numerous repeated sequences to act as a multifaceted scaffold for binding and isolating S100P, thus inhibiting S100P-associated pro-metastatic cascades (Tan et al., 2019).

Non-coding RNA activated by DNA damage has also been found to increase expression of the PI3K/AKT/mTOR pathway-related proteins. Expression of these proteins has not not considerably influenced by miR-520a-3p mimic. However, co-transfection of NORAD and miR-520 mimic has upturned the expression of these proteins. NORAD silencing has not affected expression of PI3K/AKT/mTOR pathway-associated proteins, while anti-miR-520 has enhanced expression of these proteins. Taken together, NORAD has been shown to induce the activity of PI3K/AKT/mTOR signaling through sponging miR-520 (Wan et al., 2020).

Table 1 displays summary of studies which evaluated expression of NORAD in cancer cell lines.

**TABLE 1:** Summarized results of studies which evaluated expression of NORAD in cell lines (Δ: knock-down, EMT: epithelial–mesenchymal transition).

Cancer types	Targets/regulators and signaling pathways	Cell lines	Function	Ref
Endometrial cancer	FUBP1	ISK and SPEC-2	↑ NORAD: ↓ cell growth and ↑ apoptosis	Han et al., 2020
Ovarian cancer	miR-199a-3p	SKOV3, HO8910, A2780, OVCAR-3 and IOSE80	Δ NORAD: ↓cell proliferation, invasiveness, and migration ability	Xu C. et al., 2020
	miR-608/STAT3	SKOV3, Caov3, A2780, HO-8910, OVCAR3 and HOEpiC	Δ NORAD: ↓cell viability, migration, invasiveness and ↑ apoptosis. mediating the antineoplastic impacts of physcion 8-O-b-glucopyranoside	Yang et al., 2019b
Epithelial ovarian cancer	miR-155-5p	SK-OV-3, CAOV-3, CAOV-4, OVCAR-3, HEY-T30, ES-2, SW/626 and HS832.Tc	Δ NORAD: ↓ cell proliferation and chemoresistance	Tong et al., 2019
Cervical cancer	miR-590-3p/SIP1	SiHa, HeLa, ME180, C33a, CaSki and Ect1/E6E7	Δ NORAD: ↓ cells proliferation, colony formation ability, invasion and EMT	Huo et al., 2018
Breast cancer	TGF-β pathway	MCF-7, MDA-MB-231 and MCF10A	Δ NORAD: ↓ cell proliferation, migration and invasion	Zhou et al., 2019
	YAP/TAZ-TEAD complex and S100P	MDA-MB-231, Hs578T, T47D, ZR75	Δ NORAD: ↑ cell migration and invasion	Tan et al., 2019
	miR-323a-3p/PUM1/eIF2	MCF-7, MDA-MB-231, MDA-MB-468, MDA-MB-453, T47D and MCF10A	Δ NORAD: ↓ cell viability, invasion and migration	Shi et al., 2021
Prostate cancer	miR-495-3p/TRIP13	DU145, 22Rv1, LNCaP and RWPE-1	Δ NORAD: ↑ cell apoptosis and ↓ cell proliferation, migration, and invasion	Chen et al., 2020
	miR-541-3p/PKM2	22Rv1, DU145, PC-3, C4-2B and RWPE-1	Δ NORAD: ↓ cell proliferation, migration and invasion	Hu et al., 2021
	–	LNCaP, 22Rv1, PC-3, DU145 and RWPE-1	Δ NORAD: ↓ cell proliferation, migration and ↑ cell apoptosis	Zhou et al., 2019
	miR-30a-5p/RAB11A/WNT/β-catenin pathway	PC-3, LNCap, 22RV1, DU-145 and RWPE-1	Δ NORAD: ↓ cell proliferation, invasion and EMT	Zhang and Li, 2020
Bladder cancer	–	TSSCUP, T24, 639 V and UMUC1	Δ NORAD: ↓ cells proliferation and colony formation ability	Li et al., 2018
Renal cell carcinoma	miR-144-3p/MYCN	86-O, A498, ACHN, OS-RC-2 and HK-2	↑ NORAD: ↑ cell proliferation and migratory potential	Zhao W. et al., 2020
Gastric cancer	RhoA/ROCK1 Pathway	AGS, BGC-823, HGC-27, MGC-803 and GES-1	Δ NORAD: ↑ cell apoptosis and ↓ cell proliferation and Metastatic Behavior	Yu et al., 2019
	miR-608/FOXO6	MKN28, MKN45, SGC7901, SNU-16 and GES-1	Δ NORAD: ↓ tumor growth, migration and ↑ cell apoptosis	Miao et al., 2019
	miR-214/Akt/mTOR	BSG823, MKN28, BGC803, BGC823 and GSE1	Δ NORAD: ↑ cell apoptosis and ↓ cell proliferation	Tao et al., 2019
Colorectal cancer	CAPN7 and PI3K/AKT pathway	HCT116, Caco2, Caco205, SW620, SW480 and NCM460	↑ NORAD: ↓ Cell Proliferation and Invasion	Lei et al., 2018
	–	HCT116 and SW1116	Δ NORAD: ↓ cell viability, migration and invasion	Wang et al., 2018
	miR-202-5p	SW480, HCT116 and FHC	Δ NORAD: ↓ Cell Proliferation, migration, invasion and ↑ Cellular Apoptosis	Zhang et al., 2018
	miR-203a	HCT116, SW620, SW480, HT29 and NCM460	Δ NORAD: ↓ Cell invasion	Zhao L. et al., 2020
Pancreatic cancer	miR-125a-3p/RhoA	SW1990, Capan-1, PANC-1, AsPC-1, CFPAC-1, MIAPaCa-2 and BxPC-3	Δ NORAD: ↓cell migration and invasion	Li et al., 2017
Hepatocellular carcinoma	miR-144-3p/SEPT2	Hep3B, Huh7, BEL-7402, HCCLM3 and LO2	Δ NORAD: ↓cell proliferation, colony formation and ↑ apoptosis	Tian et al., 2020
	miR-202-5p/TGF-β	SMMC-7721, Huh7, PLC/PRF/5, and Hep3B	↑ NORAD: ↑ cell proliferation, enhanced the colony construction, cell migration and invasion	Yang et al., 2019a
	miR-211-5p/FOXD1/VEGF-A axis		Δ NORAD: ↓ cell proliferation, migration and angiogenesis	Sun et al., 2021
Lung cancer	CXCR4 and CXCL12/RhoA/ROCK pathway	A549, SPC-A1, SK-MES-1 and 16HBE	Δ NORAD: ↓ Cell Proliferation, Migration and Invasion	Wu Y. et al., 2020
	miR-30a-5p/ADAM19	H460, H1299, A549, and SCLC-21H and HBE	Δ NORAD: ↓cell proliferation, migration, invasion and ↑ cell apoptosis	Wu H. et al., 2020
	YAP/TAZ-TEAD complex and S100P	H460, CL1-0, CL1-5,293T and 293FT	Δ NORAD: ↑ cell migration and invasion	Tan et al., 2019
Non-small cell lung cancer	miR-129-1-3p/SOX4	H446 and A549	Δ NORAD: resensitized to DDP (cisplatin)	Huang et al., 2020
	TGF- β	A549	Δ NORAD: ↓cell migration and EMT-like morphological changes	Kawasaki et al., 2018
	miR-656-3p/AKT1	SPC-A1, H460, H1650, A549 and HBE	↑ NORAD: ↑ cell proliferation and migration	Chen T. et al., 2019
	miR-136-5p/E2F1	A549, H1975, H1650, LK-2, H1299, H460 and HBE	Δ NORAD: ↓cell proliferation and glycolysis	Gao et al., 2019
	miR-520a-3p/PI3k/Akt/mTOR Signaling pathway	A549, H1299, H460, SK-MES-1, Calu3 and HEK293T	Δ NORAD: ↓cell Proliferation, Migration and Invasion	Wan et al., 2020
	miR-422a	A549, SK-MES-1, H1975, SK-LU-1 and 16HBE	↑ NORAD: ↑cell viability, migration, invasion and EMT	Chen et al., 2021
	miR-455/CDK14	NCI-H1650 and HCC827	Δ NORAD: ↓ proliferation ability	Wang C. et al., 2021
	miR-202-5p/P-gp	A549/DPP	Δ NORAD: ↑ cisplatin sensitivity in A549/DPP cells	Shen et al., 2020
	miR-363-3p/PEAK1 and ERK1/2 signaling pathway	H1975, H1299, A549, 95D, and H460, (HEK)-293 T, BEAS-2B and MRC5	Δ NORAD: ↓ invasion and EMT	Geng et al., 2021
Papillary Thyroid carcinoma	miR-202-5p	K1, BCPAP, TPC1 and NPA187 and HT-ori3	↑ NORAD: ↑ cell growth, invasion, migration and EMT	Chen Y. et al., 2019
Esophageal cancer	miR-26a-5p/CKS2 via MDM2/p53/Bcl2/Bax pathway	KYSE-150, ECA-109 and HEEC	Δ NORAD: ↓ cell proliferation, invasion, migration and ↑ cell apoptosis	Zhang et al., 2020
Oral squamous cell carcinoma	miR-150	Fadu, SCC-25, CAL-27, Tca8113 and Hs 680.Tg	Δ NORAD: ↓ cell proliferation	Xu F. et al., 2020
Malignant melanoma	miR-205/EGLN2	A375, WM451, SK-MEL-24, WM35 and HM	Δ NORAD: ↓ cell migration and invasion	Chen Y. et al., 2019
Glioma	AKR1B1	GSC11, M059J, U251, T98G and A735	Δ NORAD: ↓ cell proliferation, invasion, migration and ↑ cell apoptosis	Luo et al., 2020
Neuroblastoma	–	SK-N-BE, SMS-KAN, SMS-KCN, CHLA-15, CHLA-122, NBL-W, SK-N-BE, SMS-KANR, SMS-KCNR, CHLA20, CHLA-136 and NBL-WR	NORAD may be able to predict neuroblastoma outcome	Utnes et al., 2019
	miR-1443p/HDAC8	SK-N-SH, IMR-32 and HUVEC	Δ NORAD: ↓ cell proliferation, migration, invasion and ↑ apoptosis, autophagy and doxorubicin resistance	Wang et al., 2020
	Chromosomal instability	SH-SY5Y and SK-N-BE (Munschauer et al., 2018)	Δ NORAD: ↑ cell proliferation, migration and cell cycle arrest specially impaired sister chromatid cohesion and segregation	Yu et al., 2020
Osteosarcoma	miR-199a-3p	Saos-2, 143B, HOS, KHOS/240S, MG-63, U-2OS, SK-ES- and Hs755	Δ NORAD: ↓ cell proliferation and invasion	Wang et al., 2019
	miR-410-3p	HOS/DDP	Δ NORAD: ↓ cell proliferation and ↑ sensitivity to cisplatin	Xie et al., 2020
	miR-155-5p	143B, HOS, MG63, Saos-2, U2OS, hFOB and HEK-293T	Δ NORAD: ↓ cell proliferation, migration and invasion	Wang Y. et al., 2021

**FIGURE 1:**
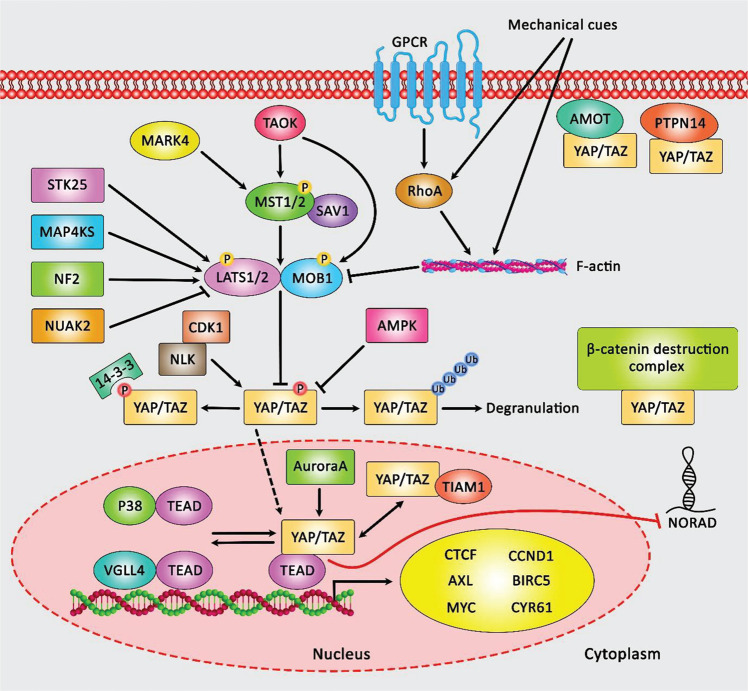
A schematic representation of the crosstalk between Hippo signaling cascade and lncRNA NORAD in lung and breast neoplasms. YAP/TAZ is mainly modulated via the canonical Hippo cascade, MST1/2-SAV1, and LATS1/2-MOB1. LATS1/2 could in turn phosphorylate YAP/TAZ and suppress its function through either ubiquitination and proteasome-mediated degradation or 14-3-3-mediated cytoplasmic sequestration. Unphosphorylated YAP/TAZ is transferred to the nucleus, where it could interact with TEAD transcription factors and trigger the expression of various target genes. LATS1/2 could be upregulated via STK25, TAOK, NF2, and MAP4KS, while being inhibited through GPCR-RHOA-mediated F-actin function mechanical cues as well as NUAK2. In addition, expression of MST1/2 is regulated by TAOK and MARK4. Expression of YAP/TAZ is also modulated in an independent manner from LATS. Besides, PTPN14 and AMOT could interact with YAP/TAZ and sequester it in the cell membrane. Expression of YAP/TAZ is downregulated via the β-catenin demolition complex or TIAM1 through a direct interaction. Phosphorylation of YAP/TAZ is triggered by CDK1, AMPK, Aurora A, NLK, and various RTKs. In addition, p38 and VGLL4 could interact with TEAD and inhibit the function of YAP/TAZ (Yamaguchi and Taouk, 2020). Mounting evidence has collectively demonstrated that the Hippo pathway transducer YAP/TAZ-TEAD complex could play an effective role in suppressing the expression level of lncRNA NORAD in both lung and breast cancers. Its downregulation correlates with enhancement of migration, invasion as well as metastasis in tumor cells (Tan et al., 2019).

Figure 1 depicts the role of Hippo cascade transducer YAP/TAZ-TEAD complex in inhibiting the expression of lncRNA NORAD in lung and breast neoplasms, and consequent attenuation of the tumor suppressor roles of NORAD in tumor cells.

**FIGURE 2:**
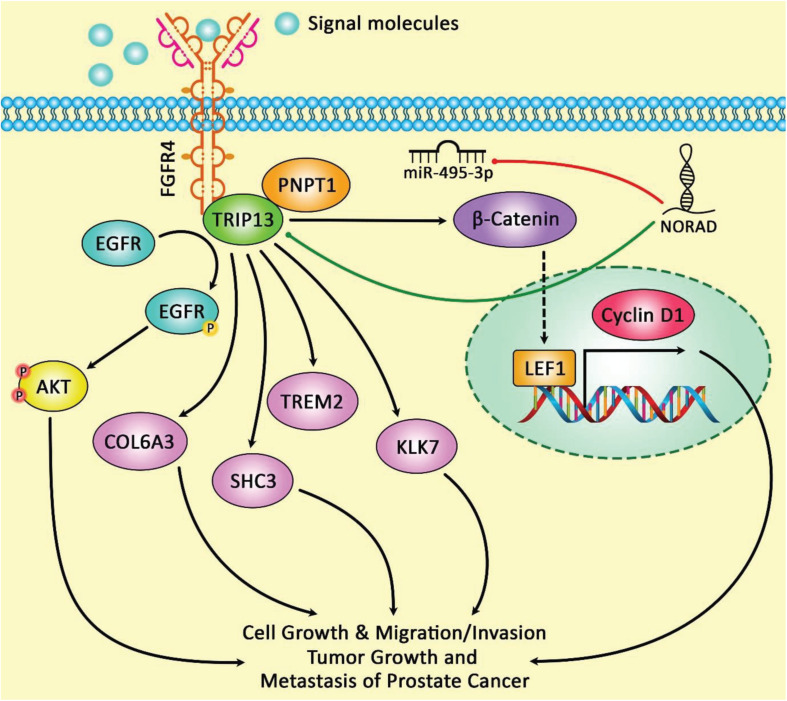
The schematic diagram of the role of lncRNA NORAD in the regulation of TRIP13 expression in prostate cancer. Overexpression of NORAD and TRIP13 and downregulation of miR-495-3p have been in prostate cancer cells. LncRNA NORAD could modulate TRIP13 expression through sponging miR-495-3p, and thereby enhancing cell proliferation, migration, and invasion as well as reducing cell apoptosis in tumor cells. In fact, NORAD could play an important role as a sponge for miR-495-3p in prostate cancer cells that attenuates the potent tumor suppressive activity of this miRNA in target cells (Chen et al., 2020).

Figure 2 demonstrates the modulation of TRIP13 expression through lncRNA NORAD indicating that TRIP13 upregulation could suppress the impacts of miR-495-3p up-regulation on the proliferation, apoptosis, migratory potential, and invasiveness of prostate cancer cells.

**FIGURE 3:**
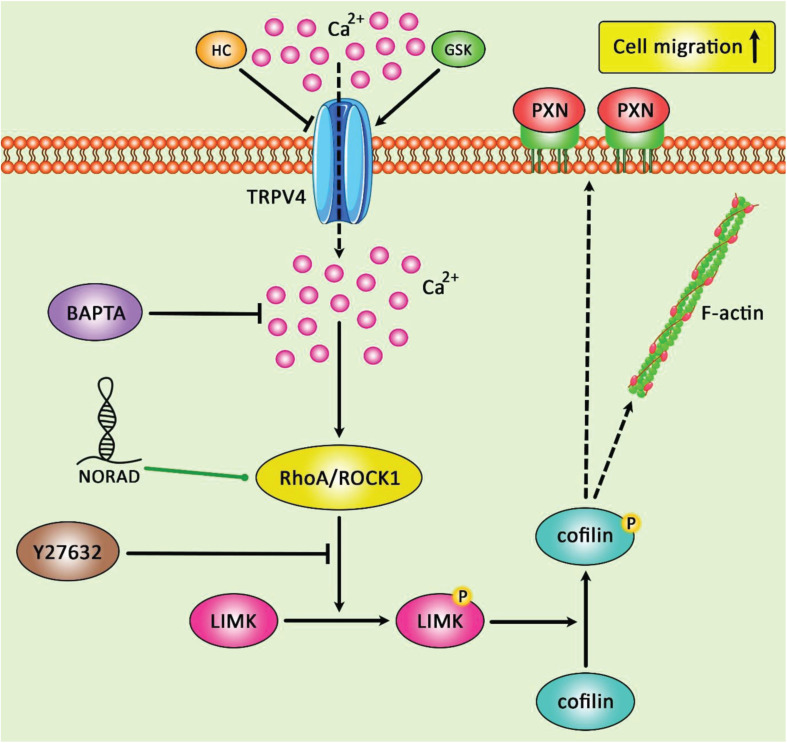
A schematic summary of the crosstalk between the RhoA/ROCK1 singling pathway and lncRNA NORAD in gastric cancer. The figure depicts the impact of RhoA/ROCK1 involved in LIMK/cofilin/TRPV4/Ca2+ pathway in gastric cancer. LncRNA NORAD could promote the expression levels of RhoA and ROCK1, and thereby enhancing cell proliferation, migration and invasiveness and reducing cell apoptosis in gastric cancer cells (Yu et al., 2019).

Figure 3 represents the oncogenic role of NORAD in gastric cancer progression via modulating the expression levels of RhoA/ROCK1.

## Human Studies

Based on the assessment of data available in The Cancer Genome Atlas (TCGA) as well as an independent cohort of patients with endometrial cancer, expression of NORAD has been decreased in endometrial cancer samples compared with normal tissue samples in association with cancer progression. Notably, has been identified as the underlying mechanism of NORAD down-regulation in these samples (Han et al., 2020). A single study in patients with colorectal cancer demonstrated down-regulation of NORAD in tumor tissues particularly in samples obtained from patients developed distant metastasis. Down-regulation of NORAD has been associated with poor patients’ outcome, advanced tumor size and TNM stage (Lei et al., 2018). Apart from these two studies, other studies have reported up-regulation of NORAD in tumoral samples compared with non-tumoral samples from the same tissue. Such over-expression has also been verified in other cohorts of patients with colorectal cancer (Wang et al., 2018; Zhang et al., 2018). Besides, expression of this lncRNA has been up-regulated in hepatocellular carcinoma (HCC) samples compared with adjacent tissues in correlation with poor overall survival (Yang et al., 2019a). Over-expression of NORAD in cervical cancer patients has been correlated with higher stage, lymph nodes and vascular involvement, and poor survival (Huo et al., 2018). Table 2 depicts the results of studies which evaluated expression of NORAD in clinical samples.

**TABLE 2:** Summarized results of studies which assessed expression of NORAD in clinical samples (OS: overall survival, ANTT: adjacent non-tumoral tissue).

Cancer types	Number of clinical samples (tissue, serum, etc.)	Expression tumor vs normal	Kaplan–Meier analysis	Univariate cox regression	Multivariate cox regression	Ref
Endometrial cancer (EC)	56 EC tissues, 54 ANTTs and 20 normal endometrial tissues, TCGA data	Down	Decreased NORAD level was correlated with poor survival in patients with EC	–	–	Han et al., 2020
Ovarian cancer (OC)	86 paired of OC tissues and ANNTs	Up	–	–	–	Xu C. et al., 2020
	56 paired of OC tissues and ANNTs	Up	–	–	–	Yang et al., 2019b
Epithelial ovarian cancer (EOC)	17 paired of EOC tissues and ANNTs	Up	–	–	–	Tong et al., 2019
Cervical cancer (CC)	47 paired of CC tissues and ANNTs	Up	NORAD upregulation was correlated with poor OS in CC patients	–	–	Huo et al., 2018
Breast cancer (BC)	21 BC tissues and 10 ANNTs	Up	NORAD upregulation was correlated with worse survival compared to the downregulation groups	–	–	Zhou et al., 2019
	44 BC tissues (subtypes: HER2, luminal A, luminal B, basal-like and triple-negative)	Up (Differentially expressed among BC subtypes)	Higher expression of NORAD in Basal-like subtype correlated with lower disease-free survival rate	–	–	Mathias et al., 2021
	108 paired of BC tissues and ANNTs	Up	–	–	–	Shi et al., 2021
Prostate cancer (PC)	30 paired of PC tissues and ANNTs	Up	–	–	–	Chen et al., 2020
	74 paired of PC tissues and ANNTs	Up	Higher NORAD expression associated with poor survival	–	–	Hu et al., 2021
	45 paired of PC tissues and ANNTs	Up	–	–	–	Zhang and Li, 2020
Bladder cancer	10 paired of BC tissues and ANNTs	Up	Over-expression of NORAD was significantly associated with worse OS	Tumor stage, histological grade, lymph node involvement, and NORAD expression were significantly associated with OS	NORAD over-expression was independent prognostic indicator for OS.	Li et al., 2018
Renal cell carcinoma (RCC)	36 paired of RC tissues and ANNTs	Up	–	–	–	Zhao W. et al., 2020
Esophageal Squamous Cell Carcinoma (ESCC)	106 paired of ESCC tissues and ANTTs	Up	NORAD upregulation was correlated with poor OS and disease-free survival in ESCC patients	Tumor differentiation, lymph node metastasis, UICC stage and NORAD expression were significantly associated with ESCC.	NORAD expression levels and UICC stage were independent prognostic factors in ESCC.	Wu et al., 2017
Gastric cancer (GC)	65 paired of GC tissues and ANTTs, GEO database	Up	NORAD upregulation was significantly correlated with worse OS in GC patients	–	–	Yu et al., 2019
	40 paired of GC tissues and ANTTs	Up	NORAD upregulation was significantly correlated with the worse prognosis of the GC patients	–	–	Miao et al., 2019
	36 paired of GC tissues and ANTTs	Up	–	–	–	Tao et al., 2019
Colorectal cancer (CRC)	80 paired of CC tissues and ANTTs	Down	–	–	–	Lei et al., 2018
	60 paired of CRC tissues and ANTTs. Serum samples from142 CRC patients, 136 normal subjects, and 71 patients with benign disorders	Up	–	–	–	Wang et al., 2018
	47 paired of CRC tissues and ANTTs	Up	Higher expression levels of NORAD suggested poorer prognosis in CRC patients compared to lower group	–	–	Zhang et al., 2018
	Serum samples from 32 CRC patients and 48 precancerous patients and 110 healthy controls	Up	–	–	–	Shaker et al., 2019
	30 paired CRC tissues and ANTTs	Up	NORAD higher expression levels associated with poor OS in CRC patients	–	–	Zhao L. et al., 2020
Pancreatic ductal adenocarcinoma (PDAC)	33 paired PDAC tissues and ANTTs	Up	PDAC patients with higher NORAD expression had shorter OS and recurrence-free survival	–	–	Li et al., 2017
Hepatocellular carcinoma (HCC)	Starbase data	Up	–	–	–	Tian et al., 2020
	29 HCC tissues and their ANTTs	Up	NORAD upregulation correlated with shorter OS rate and higher recurrence rate in HCC patients	Sex, tumor size and NORAD expression were significantly associated with OS	NORAD expression was an independent indicator of OS and recurrence after surgery.	Wu Y. et al., 2020
		Up	–	–	–	Sun et al., 2021
Lung cancer (LC)		Up	–	–	–	Wu Y. et al., 2020
	31 paired of LC tissues and ANTTs	Up	–	–	–	Wu H. et al., 2020
Non-small cell lung cancer (NSCLC)	60 paired od NSCLC tissues and ANTTs	Up	High expression levels of NORAD suggested poorer prognosis in NSCLC patients	–	–	Huang et al., 2020
	24 paired of NSCLC tissues and ANTTs	Up	–	–	–	Chen T. et al., 2019
	80 paired of NSCLC tissues and ANTTs	Up		–	–	Gao et al., 2019
	26 paired of NSCLC tissues and ANTTs	Up	–	–	–	Wan et al., 2020
	50 paired of NSCLC tissues and ANTTs	Up	–	–	–	Chen et al., 2021
		Up	Higher NORAD expression levels associated with worse prognosis in NSCLC patients	–	–	Wang C. et al., 2021
	15 paired of NSCLC tissues and ANTTs	Up	–	–	–	Geng et al., 2021
Papillary thyroid carcinoma (PTC)	40 paired of PTC tissues ANTTs	Up	–	–	–	Chen Y. et al., 2019
Oral squamous cell carcinoma (OSCC)	32 paired of OSCC tissues ANTTs	Up	Higher expression of NORAD predicted worse prognosis in OSCC patients	–	–	Xu F. et al., 2020
Malignant melanoma (MM)	62 MM tissues and 20 normal tissues	Up	–	–	–	Chen Y. et al., 2019
Neuroblastoma (NB)	38 paired of NB tissues and normal tissues	Up	–	–	–	Wang et al., 2020
	40 NB tumor specimens	Down	Lower NORAD expression correlated with poor OS and event free survival in NB patients	–	–	Yu et al., 2020
Glioblastoma (GBM)	TCGA (168 GBM tissues and 5 normal brain tissues) GTEx (105 normal brain tissues)	Up	–	–	–	Peng et al., 2020
Osteosarcoma	69 paired of tumor bone tissues and ANTTs	Up	–	–	–	Wang et al., 2019
	30 paired of osteosarcoma tissues and ANTTs	Up	–	–	–	Wang Y. et al., 2021

## Prognostic Role of NORAD in Malignancies

Apart from endometrial cancer in which up-regulation of NORAD determined good prognosis (Han et al., 2020), in other types of cancers, including cervical cancer (Huo et al., 2018), breast cancer (Zhou et al., 2019), bladder cancer (Li et al., 2018), esophageal cancer (Wu et al., 2017), gastric cancer (Yu et al., 2019), colorectal cancer (Zhang et al., 2018), pancreatic cancer (Li et al., 2017), hepatocellular carcinoma (Yang et al., 2019a), and lung cancer (Huang et al., 2020), its up-regulation was an indicator of poor survival.

## Animal Studies

**FIGURE 4:**
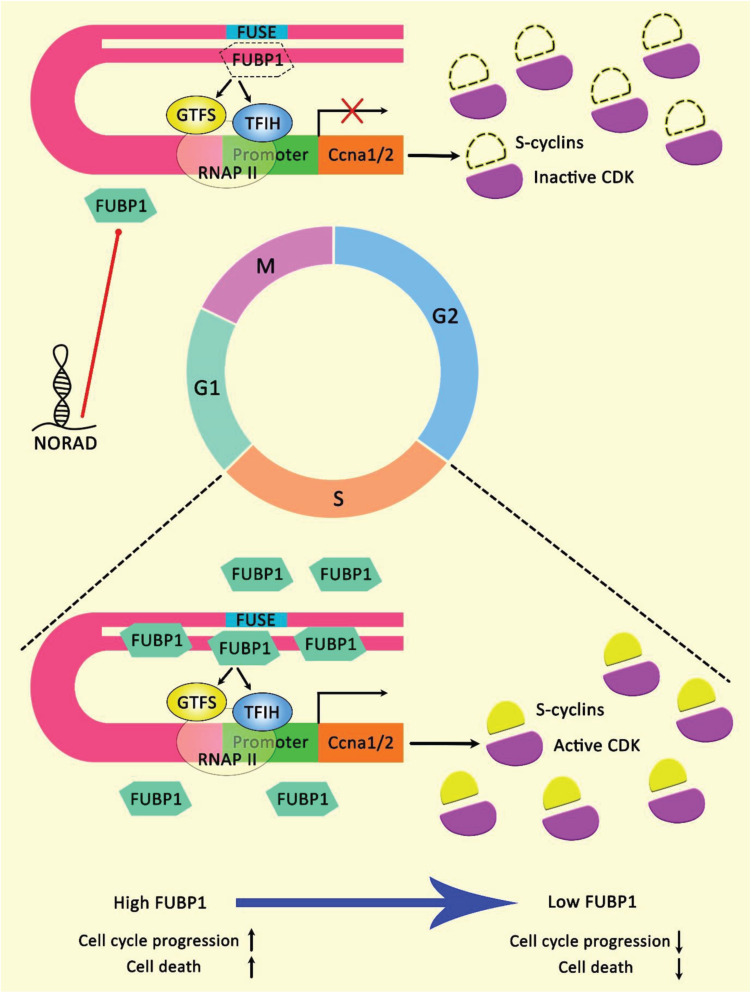
A schematic illustration of the interaction of lncRNA NORAD and FUBP1 in endometrial cancer. FUBP1 is a master transcriptional regulator of various genes via interacting with FUSE. FUBP1 protein level is upregulated in the S phase. Reducing in the expression level of FUBP1 could affect cell cycle progression, particularly in the S phase, via downregulating Ccna gene theat encodes cyclin A. Fubp1-cyclin A axis could play a crucial role in triggering various types of cancers. Heterogeneous expression patterns of Fubp1 could be seen among several cancer tissues, illustrating its multiple and sophisticated functions in cancer development (Han et al., 2020). Accumulating evidence elucidates that epigenetic inactivation of NORAD could promote cell growth and reduce apoptosis in endometrial cancer cells. NORAD/FUBP1 interaction could inhibit FUBP1 nuclear localization, and thereby downregulating the recruitment of FUBP1 on promoters of target pro-apoptotic genes, triggering apoptosis in tumor cells (Han et al., 2020).

Endometrial cancer is among few cancer types in which NORAD exerts anti-oncogenic effects. Such effects have been verified in animal models since NORAD silencing has enhanced tumor growth in the xenograft model. On the other hand, over-expression of FUBP1-binding fragment of NORAD has attenuated tumor growth in this model (Han et al., 2020). Figure 4 illustrates the effect of lncRNA NORAD binding with FUBP1 in endometrial cancer cells.

Apart from this study, other *in vivo* studies have shown the role of NORAD in enhancement of tumor progression in animal models. For instance, NORAD increases the growth of neuroblastoma tumors in animal models via miR-144-3p/HDAC8 axis (Wang et al., 2020). Moreover, growth of osteosarcoma tumors in animals has been attenuated by NORAD silencing in the implanted cells (Wang et al., 2019). Further studies in malignant melanoma, cervical cancer, breast cancer and lung cancer supported oncogenic effects of NORAD in xenograft models. Table 3 recapitulates the results of studies which evaluated the role of NORAD in the development of cancer in animal models.

**TABLE 3:** Outline of studies which assessed function of NORAD in animal models (Δ: knock down or deletion).

Cancer types	Animal models	Function and comment	Ref
Endometrial cancer	Female BALB/c nude mice	Δ NORAD: ↑ Tumor growth	Han et al., 2020
Epithelial ovarian cancer	Adult female athymic nude mice	Δ NORAD: ↓ Tumor volume	Tong et al., 2019
Cervical cancer	Athymic BALB/c mice	Δ NORAD: ↓ Tumor volume and weight	Huo et al., 2018
Breast cancer	Female BALB/c mice	Δ NORAD: ↓ Tumor Growth	Zhou et al., 2019
	male BALB/c-nu/nu nude mice	Δ NORAD: ↓ Tumor Growth	Shi et al., 2021
Prostate cancer	BALB/c-nu mice	Δ NORAD: ↓ bone metastasis	Hu et al., 2021
	BALB/C nude mice	Δ NORAD: ↓ Tumor volume and weight	Zhang and Li, 2020
Gastric cancer	BALB/c nude mice	Δ NORAD: ↓ Tumor volume	Tao et al., 2019
Colorectal cancer	Male BALB/c nude mice	Δ NORAD: ↓ Tumor Growth	Zhang et al., 2018
Hepatocellular carcinoma	Mice	Δ NORAD: ↓ Tumor Growth	Tian et al., 2020
	nude mice	Δ NORAD: ↓ Tumor weight	Yang et al., 2019a
Lung cancer	Male athymic nude BALBC/c	Δ NORAD: ↓ Tumor Growth	Wu Y. et al., 2020
Non-small cell lung cancer	Mice	Δ NORAD: ↓ Tumor weight and volume and metastasis	Wan et al., 2020
Malignant melanoma	Male BALB/c-nu/nu	Δ NORAD: ↓ Tumor Growth	Chen Y. et al., 2019
Neuroblastoma	Flank of mice	Δ NORAD: ↓ Tumor Growth	Wang et al., 2020
Osteosarcoma	Nude female BALB/c mice	Δ NORAD: ↓ Tumor Growth	Wang et al., 2019

## Discussion

Numerous studies have evaluated the role of NORAD in the development of cancer. With the exception of two studies in endometrial and colorectal cancer, other studies indicate the oncogenic role of this lncRNA in diverse cancer types. Several miRNAs such as miR-199a-3p, miR-608, miR−155−5p, miR-590-3p, miR-495-3p, miR-608, miR-202-5p, miR-125a-3p, miR-144-3p, miR−202−5p, and miR-30a-5p have been recognized as targets of NORAD in different cancer cell lines. In addition, NORAD has interactions with cancer-related pathways such as STAT, TGF-β, Akt/mTOR, and PI3K/AKT pathway. The role of NORAD in activation of TGF-β has been verified in different cancers, namely hepatocellular carcinoma, breast cancer and lung cancer. This function is implicated in the enhancement of EMT features and invasive properties of cancer cells. Therefore, in addition its role in the initiation of cancer possibly through influencing genomic stability, NORAD partakes in the progression of cancer through enhancement of EMT. In addition, NORAD has a role in the modulation of response of cancer cells to a number of chemotherapeutic drugs such as doxorubicin and cisplatin (Huang et al., 2020; Wang et al., 2020).

*In vivo* studies in xenograft models of ovarian, cervical, breast, gastric, colorectal, liver and lung cancers as well as neuroblastoma and osteosarcoma have shown the efficacy of NORAD-targeting therapeutic options in reducing tumor burden. Therefore, this lncRNA is a putative target for treatment of cancer.

The prognostic value of NORAD has been verified in diverse cancer types such as lung, liver, pancreatic, colorectal, breast and cervical cancers where over-expression of this lncRNA was correlated with poor survival. Based on the significant difference in expression of this lncRNA between cancerous and non-cancerous tissues, assessment of its expression might provide a diagnostic tool for cancer. However, the sensitivity and specificity of this marker should be assessed in diverse cancer types. Moreover, assessment of its expression in body fluid such as blood, serum and urine might help in the development of non-invasive diagnostic methods. The latter possible application of NORAD has not been assessed yet.

The data presented above indicate up-regulation of NORAD in almost all types of neoplasm. Moreover, functional studies have shown the pro-proliferative, pro-migratory, and pro-metastatic abilities of NORAD. Collectively, NORAD in an oncogenic lncRNA in most tissues and a possible target for inventions against cancer. Future investigations are required to support its application as diagnostic marker in the clinical settings.

## Author Contributions

MT and SG-F wrote the draft and revised it. ND, TA, BH, and AA collected the data and designed the tables and figures. All authors read and approved the submitted version.

## Conflict of Interest

The authors declare that the research was conducted in the absence of any commercial or financial relationships that could be construed as a potential conflict of interest.

## Publisher’s Note

All claims expressed in this article are solely those of the authors and do not necessarily represent those of their affiliated organizations, or those of the publisher, the editors and the reviewers. Any product that may be evaluated in this article, or claim that may be made by its manufacturer, is not guaranteed or endorsed by the publisher.
